# GlideScope Use improves intubation success rates: an observational study using propensity score matching

**DOI:** 10.1186/1471-2253-14-101

**Published:** 2014-11-05

**Authors:** James W Ibinson, Catalin S Ezaru, Daniel S Cormican, Michael P Mangione

**Affiliations:** Department of Anesthesiology, VA Pittsburgh Healthcare System and Department of Anesthesiology, University of Pittsburgh School of Medicine, Pittsburgh, PA USA; Department of Anesthesiology, University of Pittsburgh Medical Center, Pittsburgh, PA USA

**Keywords:** Airway management, Direct laryngoscopy, Difficult intubation

## Abstract

**Background:**

Rigid video laryngoscopes are popular alternatives to direct laryngoscopy for intubation, but further large scale prospective studies comparing these devices to direct laryngoscopy in routine anesthesiology practice are needed. We hypothesized that the first pass success rate with one particular video laryngoscope, the GlideScope, would be higher than the success rate with direct laryngoscopy.

**Methods:**

3831 total intubation attempts were tracked in an observational study comparing first-pass success rate using a Macintosh or Miller-style laryngoscope with the GlideScope. Propensity scoring was then used to select 626 subjects matched between the two groups based on their morphologic traits.

**Results:**

Comparing the GlideScope and direct laryngoscopy groups suggested that intubation would be more difficult in the GlideScope group based on the Mallampati class, cervical range of motion, mouth opening, dentition, weight, and past intubation history. Thus, a propensity score based on these factors was used to balance the groups into two 313 patient cohorts. Direct laryngoscopy was successful in 80.8% on the first-pass intubation attempt, while the GlideScope was successful in 93.6% (p <0.001; risk difference of 0.128 with a 95% CI of 0.0771 – 0.181).

**Conclusion:**

A greater first-attempt success rate was found when using the GlideScope versus direct laryngoscopy. In addition, the GlideScope was found to be 99% successful for intubation after initial failure of direct laryngoscopy, helping to reduce the incidence of failed intubation.

## Background

Unsuccessful direct laryngoscopy for orotracheal intubation occurs with an incidence reported to be as high as 0.3% to 0.43% in two large studies [1, 2]. Various alternatives to standard direct laryngoscopy are often deployed when a potential “difficult airway” is identified [3] or when conventional laryngoscopy fails. Over the past several years, video laryngoscopic devices like the GlideScope (Verathon, Inc., Bothell, WA) have come to the forefront of direct laryngoscopy alternatives. These devices do not require line of sight visualization of the larynx; instead videochip/camera technology projects a view of the patient’s larynx onto a video screen. The latest report from the American Society of Anesthesiologists Task Force on the management of the difficult airway [4] even includes the consideration of video laryngoscope devices as an initial approach to intubation.

Several studies have addressed the aspects of video laryngoscope use. Aziz et al. reported retrospective data of a very large number of intubations (71,570 intubations including 2,004 GlideScope uses) demonstrating the GlideScope’s high success rates as a primary device and a rescue device (98% and 94%, respectively), and providing insight into the incidence of major complications with the device (0.3%) [5]. Simulation-based studies describe greater intubation success rates using video laryngoscopy when compared to direct laryngoscopy [6, 7], although the applicability of these to real-world practice could be questioned. Prospective studies that describe video laryngoscope use in patients have shown a better Cormack-Lehane view than direct laryngoscopy in certain scenarios [3, 8, 9], however video laryngoscope intubations appear to take longer to perform [6, 10].

This study seeks to contribute to the literature by addressing video laryngoscope use in routine airway management and anesthesiology practice within an active teaching hospital via prospective investigation. The practitioners were free to use the intubation technique they considered most appropriate and were directed to specify the reason if video laryngoscopy was employed. We hypothesized that the first pass success rate with the GlideScope would be higher than the success rate with direct laryngoscopy regardless of the experience of the operator. Our primary aim was to collect data to confirm the aforementioned hypotheses, using first pass success as a measurement of intubation ease. Secondary aims included the identification of the patient morphologic factors that influenced anesthesiologists to select the GlideScope because of the potential for a “difficult airway” and the determination of complication rates, as we further hypothesized that the complication rates between the two would be no different.

## Methods

This study was reviewed and approved by the institutional review board of the Veterans Administration of Pittsburgh Health Care System. The VAPHCS is a tertiary care/transplant center for a wide variety of adult surgical patients with the exception only of Level I trauma. All patients over the age of 18 undergoing surgery that required intubation in the operating room were enrolled in this non-randomized, prospective observational study. Written informed consent was not required as no alterations to patient care were involved according to the institutional review board. Information for all intubations was recorded over the period of July 1, 2010 to November 1, 2011. Prior to the initiation of the study, the form was presented to the anesthesia providers at a monthly staff meeting, and proper completion of the form was emphasized at subsequent meetings.

The GlideScope device has been in use in this facility since 2006. Practitioners performing the intubation included medical students, student nurse anesthetists, anesthesiology residents at all levels of training, certified registered nurse anesthetists, and attending anesthesiologists. Our facility consists of ten sites where general anesthesia may be provided; at least two GlideScope devices were available every day to the practitioners during this study. Additionally, our supply of cleaned, reusable blades was maintained at a level that ensures GlideScope blades are available for each intubation over the course of a clinical day. There were no recorded cases where the GlideScope could not be used due to a lack of availability. Every intubation was supervised by one of 12 board-certified attending anesthesiologists (average experience 11.5 years post residency); restrictions or limitations on practitioner level for each intubation were not specified.

### Outcome measures

The primary outcome measure was “first pass” successful intubation, although data were also collected for any and all subsequent attempts. There were no limitations on the number of laryngoscopic attempts with any device, and actions to be taken in the event of intubation failure were not specified. For the purposes for the study, an intubation attempt was defined as the placement of a laryngoscope into the patient’s mouth. Removal of the blade or giving the laryngoscope handle to another practitioner was considered an “intubation failure”. Secondary outcomes included the identification of the patient characteristics that lead to a choice of the video laryngoscope because of the potential for a “difficult intubation”, and complication rate and type.

All airway assessments were performed by the anesthesiologist supervising/performing the intubation. The Samsoon and Young modification of the Mallampati score [11] was graded on the standard 1 to 4 scale with a picture printed on each intubation form for reference. Subject position and/or phonation during the exam were not specified. Cervical Range of Motion (CROM) was assessed and categorized as either “Normal” or “Decreased”. The presence of a beard, normal mouth opening, presence or absence of teeth, thyromental distance (TMD), gender, height, weight, history of being a “difficult intubation”, case type, and location were also graded on a binary scale. A four-point scale was used for age. The categorization of the above was left to the anesthesiologist; i.e., a numerical cut-off was not provided for these measures.

The details regarding the intubation itself were recorded post-attempt. Attempt success was defined as confirmed passage of the endotracheal tube beyond the vocal cords. The practitioner level (Attending Anesthesiologist, Certified Registered Nurse Anesthetist, Anesthesiology Resident, or Other) was recorded utilizing a four point scale. The device was recorded as the Macintosh or the Miller blade (with these devices combined for analysis into a “Direct Laryngoscopy” (DL) group), the GlideScope (GS) group, the Storz C-MAC (using a Macintosh blade, Karl Storz Endovision, Inc., Charlton, MA), or the bronchoscope; however only DL versus GS comparisons were performed due to low numbers in the other two groups that prohibited matching. The presence or absence of a stylet, and its type if present, was also recorded but its use during intubation was not required.

Finally, the presence and detail of any complications immediately identified by the anesthesia team were recorded. Via survey of our staff we identified a comprehensive list of our most common complications. We also included a category named “Other” where the provider could record any events that did not fit into one of our nine defined complication categories.

### Statistical analysis

Based on the literature, the nature of our patient population, and the ratio of experienced practitioners to trainees, we assumed that the overall first pass intubation success rate for the DL group at our training institution would be approximately 80%. Targeting a power of 0.8, an alpha of 0.05, and a detected difference of 5%, each group (GS and DL) would require 945 subjects. It was estimated that approximately 2000 intubations are completed each year at our facility, so to ensure adequate study power, the decision was made to collect data on all intubations for 16 months. The data sheets for the first month were used to familiarize the staff with completing the form to establish a uniform approach. These results were reviewed with the staff weekly to identify any weaknesses in data collection and assure uniformity of data recording. These data were then discarded.

The data from the intubation forms was entered into two Microsoft Excel (v2007) spreadsheets independently by two of the authors (DSC and JWI). These spreadsheets were then compared and all discrepancies resolved by referring to the original data forms. Questions of ambiguity were resolved via group discussion with all authors. This corrected and verified database was then transferred to IBM SPSS (v.18).

Descriptive statistics were obtained for all morphologic/demographic data. Categorical data (first pass success rates, the demographics and morphologic traits between the groups, etc.) were tested with a Fisher’s exact test (for 2x2 tables) or a Chi-square, both with two-sided tails. Continuous variables were compared with a two-sample t-test. An overall p-value of 0.05 was used for significance and was Bonferroni corrected for multiple comparisons for the morphologic traits.

A binary logistic regression against the “expected difficulty with DL” label for the GS group was performed with all of the morphologic characteristics. The model was built with a likelihood ratio based forward selection procedure and stability was verified with a backward selection model. Results were examined using the calculated odds ratios with 95% confidence intervals (CI).

A propensity scoring technique [12] (using an SPSS add-in)^a^ was then utilized in this analysis. Matching was done on the probability of the first-pass attempt being performed with the GlideScope, based on the predictors identified as significant in the logistic regression described above. As will be described, these included Mallampati class, CROM, normal mouth opening, presence or absence of teeth, and weight. Using these, a propensity score was calculated that ranged from 0 to 1, representing the likelihood that the GlideScope would be used on the first intubation attempt. Only subjects with complete data sets were used for the logistic regression and the propensity scoring analysis. 313 of the 643 patients in the GlideScope first-pass group were able to be matched via a nearest neighbor algorithm (with one-to-one matching and no caliper definition) to 313 patients in the DL group, and their results were compared. Patients with a known history of difficult intubation were excluded from matching as their numbers were low in the DL group. Patients not in the GS or DL groups were also excluded.

## Results

There were a total of 3384 patients in the entire study, with 3139 of these in either the DL or GS groups. Devices not included in this study were used for the other intubations. The GlideScope was used as the first-pass device in 643. On those sheets that circled a reason for GlideScope use for the first pass attempt, 329 were for “Expected Difficulty with DL” and 113 were for “Training”. Given that the typical patient at a Veterans Administration hospital is that of older male patients, the patient characteristics for the DL and GS were consistent with expectations and are given with p-values for the comparisons in Table 1. With 10 compared characteristics, a p-value of 0.005 would be needed for significance after Bonferroni correction. Mallampati class, CROM, mouth opening, presence of dentition, weight, and a past history of difficult intubation were all significantly worse for the GS group.

**Table 1:** **Patient characteristics broken down for Direct Laryngoscopy (DL)** ***versus*** **GlideScope (GS) groups**

		DL	GS
**Mallampati Class**		**p-value <0.001**	
	1	27%	11%
	2	60%	51%
	3	13%	34%
	4	0%	4%
**Cervical Range of Motion**		**p-value <0.001**	
	Normal	94%	67%
	Decreased	6%	33%
**Beard**		p-value =0.0731	
	Yes	24%	28%
	No	76%	72%
**Mouth Opening**		**p-value <0.001**	
	Normal	95%	78%
	Decreased	5%	22%
**Edentulous**		**p-value <0.001**	
	Yes	33%	21%
	No	67%	79%
**Thyromental Distance**		**p-value <0.001**	
	< 3 Fingerbreadths	10%	17%
	> 3 Fingerbreadths	90%	83%
**Sex**		p-value =0.2299	
	Male	94%	93%
	Female	6%	7%
**Body Mass**		**Wt. p-value <0.001**	
	Weight (kg)	91.4	98.3
	Height (cm)	176.7	176.7
**Age**		p-value =0.014	
	< 25	1%	0%
	25-44	11%	12%
	45-64	53%	59%
	> 65	35%	30%
**History of Difficult Intubation**		**p-value <0.001**	
	Yes	1%	12%
	No	99%	88%

P-values in bold are significant. Percentages may not add to 100% secondary to rounding and missing data.

Given the above differences in the two groups, the 329 GlideScope uses for “Expected Difficulty with DL” were compared to the intubations performed in the DL group to determine the traits that lead to the expected difficulty distinction and subsequent GlideScope choice via a binary logistic regression. Seven traits were found to be significantly associated: increasing Mallampati score (odds ratio = 2.184, 95% C.I. of 1.680 – 2.839 for each increase in Mallampati score), decreased CROM (odds ratio = 6.957, 95% CI of 4.779 – 10.126), presence of teeth (odds ratio = 1.875, 95% CI of 1.264 – 2.781), decreased mouth opening (odds ratio = 3.178, 95% CI of 2.020 – 5.000), increased weight (odds ratio = 1.014, 95% CI of 1.007 – 1.021), a case type of “emergent” (odds ratio = 1.858, 95% CI of 1.093 – 3.158), and a history of a difficult airway (odds ratio = 17.048, 95% CI of 8.952 – 32.466).

**Figure 1:**
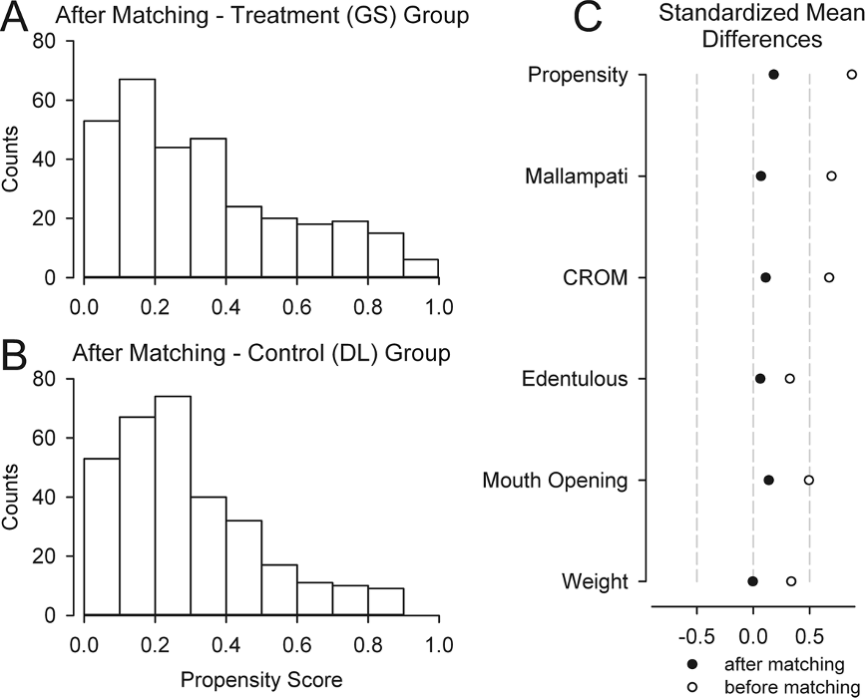
**Using the patient characteristics shown to predict GlideScope use, a propensity scoring algorithm was used to match the groups. A)** The distribution of scores in the GlideScope group. **B)** The Control (DL) group’s raw data showed a strong skewing toward lower propensity scores as expected (not shown), but the displayed propensity score distribution after matching was similar to the GS group’s. **C)** The standardized differences (Cohen’s d) were reduced for all included variables.

**Figure 2:**
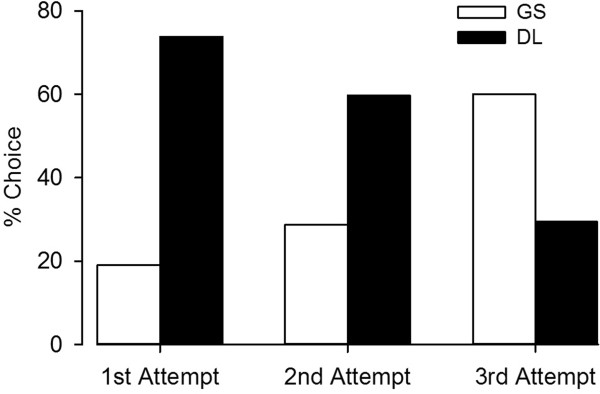
**Graph illustrating the percent of intubations using each of the intubation devices as a function of attempt.** With each successive intubation attempt, the use of a video laryngoscope increased.

A propensity score analysis based on the above traits was then performed in an attempt to balance the groups. Cases with an emergent case type or history of a difficult airway were not used as too few were present in the DL group. The propensity score technique pairs a patient in the DL group with one in the GS group that had the same probability (based on their characteristics) of being in the GS group. A total of 626 subjects were matched, 313 each in the GS and DL groups, within the prescribed limits. An excellent distribution matching resulted (Figure 1) and the patient characteristics for the DL and GS arms are given in Table 2, showing equally matched groups. Of the 313 DL patients, 253 (80.8%) were successfully intubated on the first-pass. In the GS group however, 293 of the 313 (93.6%) had first-pass success (p <0.001; risk difference of 0.128 with a 95% CI of 0.0771 – 0.181). Practitioner level was not found to significantly affect the success rate for the 626 patients in the propensity score analysis as the majority of intubation attempts were done by certified registered nurse anesthetist in both groups (51.4% for the GS group and 53.4% for the DL group).Of the 2496 patients initially in the DL group, a GlideScope was used for “rescue” purposes in 86 (3.4%, with a 95% confidence interval lower limit of 2.8%) for one of the subsequent attempts. 85 of these rescue attempts were eventually successful with the GlideScope. The percentage of intubations attempted with the GlideScope increased with each successive attempt (Figure 2). There were 13 patients that required more than 3 attempts. A single patient was not successfully intubated on the fourth attempt (three failed attempts with direct laryngoscopy, then one failed attempt with a GlideScope). This patient had a severe coagulopathy and the decision was made to use a flexible bronchoscope in order to avoid further trauma and potential bleeding from the continued use of a rigid device. All 3384 patients in the study were successfully intubated. If one counts the above mentioned conversion to a flexible fiberoptic bronchoscope as a “laryngoscopic failure”, then we observed an overall failure rate of 0.029% (1/3384, with a 95% confidence interval upper limit of 0.17%).

**Table 2:** **Patient characteristics for Direct Laryngoscopy (DL) and GlideScope (GS) groups that resulted** ***after propensity score matching***

		DL	GS
**Mallampati Class**		p-value =0.535	
	1	10%	9%
	2	52%	51%
	3	36%	36%
	4	2%	4%
**Cervical Range of Motion**		p-value =0.185	
	Normal	66%	60%
	Decreased	34%	40%
**Beard**		p-value =0.038	
	Yes	80%	73%
	No	20%	27%
**Mouth Opening**		p-value =0.088	
	Normal	80%	74%
	Decreased	20%	26%
**Edentulous**		p-value =0.498	
	Yes	23%	20%
	No	77%	80%
**Thyromental Distance**		p-value =0.084	
	< 3 Fingerbreadths	10%	14%
	> 3 Fingerbreadths	90%	86%
**Sex**		p-value =0.713	
	Male	96%	95%
	Female	4%	5%
**Body Mass**		Wt. p-value =0.955	
	Weight (kg)	100.2	100.1
	Height (cm)	177.4	176.6
**Age**		p-value =0.176	
	< 25	0%	0%
	25-44	8%	10%
	45-64	56%	61%
	> 65	35%	29%

Percentages may not add to 100% secondary to rounding and missing data.

Table 3 details the complications recorded during the study. Comparison between the GS and DL groups showed that the patient complications within the GS group were significantly greater only in regards to mucosal injury (1.5% vs 0.5%) and the ability to visualize the cords but an inability to pass the endotracheal tube. While the percentage of esophageal intubations was higher in the DL group (0.9% vs 0.3%), it did not reach statistical significance.

**Table 3:** **Overall numbers (across all attempts) of the complications recorded for each of the techniques**

Complication	GS	DL	p-value
Able to visualize the vocal cords but unable to pass the endotracheal tube	29 (3.59)	43 (1.57)	**< 0.001**
Arrythmia	0	1	1.000
Dental Injury	1	6	1.000
Esophageal Intubation	2	24	0.095
Hypoxemia	2	12	0.748
Mucosal Injury	12 (1.49)	15 (0.55)	**0.010**
Regurgitation	0	2	1.000
Other	7	23	
None	737	2585	
Total Number of Attempts with Device	808	2736	

The table lists the absolute numbers; for comparisons with significant P-values the percentage with regards to the total attempted with that device is given in parentheses.

## Discussion

A greater first-attempt success rate was found when using the GlideScope versus direct laryngoscopy. In addition, the GlideScope was found to be 99% successful for intubation after initial failure of direct laryngoscopy. In our study, however, the GlideScope’s success was found to be at the expense of a higher rate of minor mucosal injury. Although there are a multitude of clinical situations and patient-specific anatomical features that are associated with possible difficult airway, this study identifies seven specific items that were associated with the choice to utilize the GlideScope for the patient’s first intubation attempt.

This study adds to the literature on video laryngoscopy studies [3, 10, 13–15] in its size, its prospective observational design, and its focus on the everyday use of rigid direct and rigid video laryngoscopy in combination. We did not exclude any patients from our observational study, which includes a broad array of tertiary care level patients that may have been left out of other prospective studies (e.g.: emergent/rapid sequence induction cases, those with cervical mobility/immobility concerns). Moreover, our study’s data reflect the performance of all providers performing laryngoscopy – including medical and nurse anesthesia students, respiratory therapists, all levels of anesthesiology residents, Certified Registered Nurse Anesthetists, and Attending Anesthesiologists. This variable did not affect the results as the groups were well matched on this variable for the propensity score analysis. Future subgroup studies focusing on laryngoscopy skill would necessitate an increased number of subjects. While the proportion of subjects in each arm was not what we assumed for our power analysis, statistical significance was reached for our primary aim.

Greater intubation success with the GlideScope is of significant clinical importance in daily anesthetic practice. Furthermore, our study further supports the use of the GlideScope as a rescue device when direct laryngoscopy is unsuccessful. Aziz et al. reported a 94% successful rescue rate in a retrospective study [5]. The GlideScope was heavily favored as a rescue device in our institution after initial direct laryngoscopy failure as well, possessing a 98.8% rescue rate. Our successful intubation rate across the non-bronchoscope groups was 99.97%. This is an impressive rate – by incorporating the GlideScope, we were able to reduce our incidence of failed intubation to near zero. The ASA Difficult Airway Algorithm provides a framework for airway management, including a pathway for cases in which initial laryngoscopy does not succeed [4]. There is a wide array of devices available to assist in intubation efforts in the setting of failed laryngoscopy. At present it is unclear which video laryngoscope device is best for use after direct laryngoscopy failure. With the findings in this study and others involving video laryngoscopy [14, 15], further iterations of the pathway may require more attention to the GlideScope and video laryngoscopy in general.

Complications associated with intubation (or with the inability to intubate) can cause significant patient harm [16, 17]. Our study found that while most of the complication rates were equivalent, there was an statistical increase in mucosal injuries in the GS group. Injuries of this type were minor and included abrasions within the oropharynx and lip lacerations. These are well described in existing literature. We did not see any soft tissue perforations, which have been noted with GlideScope use [18, 19]. Also increased was the percentage of intubations that failed secondary to an inability to pass the endotracheal tube through the vocal cords, although the cords were visible. This, too, is often cited with the GlideScope, and has been substantially addressed within the literature. We should note, however, that ten of the GlideScope intubations mentioned here were performed without the manufacturer-recommend stylet, and that this may have altered the number of “inability to pass the tube” complications. Because of the rare nature of more serious complications, we are unable to comment further.

We found seven factors that were significantly associated to the anesthesiologists’ choice for using the GlideScope as the device for first attempt at intubation. There are a multitude of patient factors that raise an anesthesiologist’s suspicion for a possible difficult intubation; these might range from an evidence-based scoring system for prediction of difficult intubation (e.g.: Simplified Airway Risk Index [20]) to an overall gestalt based on an examination of the patient’s history and physical characteristics. A 2005 meta-analysis of the existing literature identified the combination of Mallampati score and thyromental distance as the most accurate predictor of difficult intubation using direct laryngoscopy in non-obese patients [21], although it still had room for improvement with an area under of the curve for the receiver operating characteristic of only 0.8. We did not measure, and our study does not present, specific quantitative data regarding these factors because we feel the method we employed represents routine anesthesiology practice, in which the overall impression is used more than specific numeric measurements of elements of the airway exam.

There are limitations to our study. We did not randomize our group of patients. Propensity score matching was used in an attempt to account for this weakness. Furthermore, randomization would have prevented the investigation of the patient factors associated with an anesthesiologist’s choice to use the GlideScope for the initial intubation attempt. Additionally, our study used categorical descriptions for airway exam elements that were initially described in continuous terms to predict difficult visualization and/or intubation with direct, line-of-sight laryngoscopic approaches. Examples include CROM, mouth opening, and Mallampati score. The results might vary if continuous numerical parameters were used. The ability to generalize our study is limited by the predominately older male demographics of the veteran population examined, and similar large prospective studies should be done in other settings. Finally, a consensus on the parameters defining “difficult GlideScope visualization” has not yet been reached, although the literature suggests that a high Cormack and Lehane grade, high upper lip bite, and short sternothyroid distance [22] and altered neck anatomy [5] are potential indicators. While our study did include the otolaryngology surgeries that occurred in our institution, a focused effort in a center with a large number of complicated otolaryngology cases may be needed to determine the parameters that would predict difficult visualization/intubation with the GlideScope.

## Conclusion

The use of the GlideScope is associated with greater first pass intubation success than direct laryngoscopy even though the GlideScope was used with more frequency in patients with predictors of difficult intubation. Furthermore, it was shown to be a particularly effective choice for intubation success after failed direct laryngoscopy. The use of the GlideScope did result in a greater proportion of minor complications compared to direct laryngoscopy, particular for soft tissue lacerations.

## Endnote

^a^Thoemmes, F. Propensity Score Matching in SPSS. Available at http://sourceforge.net/projects/psmspss/files/. Accessed on 11/13/2012.

## Abbreviations

CROM: Cervical range of motion
TMD: Thyromental distance
DL: Direct laryngoscopy
GS: GlideScope
CI: Confidence interval.
